# Supplementary material for: Effect of an Environment Friendly Heat and Relative Humidity Approach on γ-Aminobutyric Acid Accumulation in Different Highland Barley Cultivars
Source: Foods. 2022 Feb 25;11(5):691. doi: 10.3390/foods11050691 (PMC8908996; doi:10.3390/foods11050691)
Supplement: Supplementary file 1 [file foods-11-00691-s001.zip › Supplementary Materials.pdf]

Table S1. Description of highland barley cultivars.

Symbol	Cultivar	Seed color	Row type	Symbol	Cultivar	Seed color	Row type
YT1	QTB13	Yellow	Two-row	PS1	GMZ	Purple	Six-row
YT2	QTB25	Yellow	Two-row	PS2	LZH	Purple	Six-row
YT3	1127	Yellow	Two-row	PS3	LZZ	Purple	Six-row
YT4	1128	Yellow	Two-row	PS4	XZDM5658	Purple	Six-row
YS1	ZQ25	Yellow	Six-row	PS5	XZDM5191	Purple	Six-row
YS2	XL22	Yellow	Six-row	PS6	XZDM5122	Purple	Six-row
YS3	ZQ2000	Yellow	Six-row	PS7	XZDM5633	Purple	Six-row
YS4	ZQ320	Yellow	Six-row	PS8	XZDM5505	Purple	Six-row
YS5	ZQ13	Yellow	Six-row	PS9	XZDM5230	Purple	Six-row
YS6	ZQ27	Yellow	Six-row	PS10	XZDM5532	Purple	Six-row
YS7	XZDM4630	Yellow	Six-row	PS11	XZDM327	Purple	Six-row
YS8	XZDM4438	Yellow	Six-row	PS12	XZDM339	Purple	Six-row
YS9	XZDM4550	Yellow	Six-row				

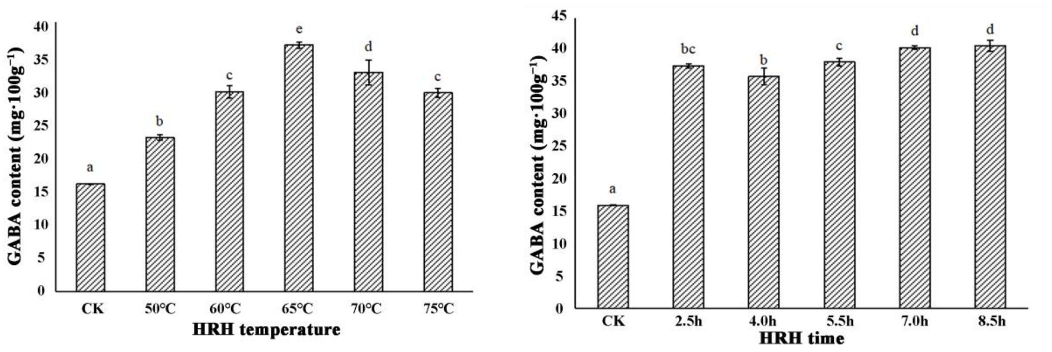

(**a**) (**b**)

Figure S1. Effect of heat and relative humidity (HRH) treatment on GABA content. (a) The effect of HRH temperature on GABA content; (b) the effect of HRH time on GABA content. Different letters denote significant difference among different HRH treatment conditions at the level $p < 0.05$. According to the capacity and efficiency of GABA accumulation, 65℃ and 2.5h were used as the treatment condition in this experiment.
